# Supplementary material for: Perceived barriers to timely treatment initiation and social support status among women with breast cancer in Ethiopia
Source: PLoS One. 2021 Sep 13;16(9):e0257163. doi: 10.1371/journal.pone.0257163 (PMC8437283; doi:10.1371/journal.pone.0257163)
Supplement: S1 Questionnaire — (PDF) [file pone.0257163.s002.pdf]

S1 Questionnaire

English and Amharic version of the questionnaire used for data collection within the study "Perceived barriers to timely treatment initiation and social support status among women with breast cancer in Ethiopia"

English Version

Consent Form

Dear participant,

My name is (…). I am a data collector for the study conducted by Bethel Teshome from Addis Ababa University College of health science and school of public health. The aim of the interview is to assess the barriers to initiate treatment among breast cancer patients in Tikur Anbessa Oncology unit. You are selected to be a part of the study as a sample population to participate in this interview. The information obtained from the study will only be used for research purpose. Your name will not be included in the questionnaire and all the information you will provide will be kept strictly confidential. There is no financial or other benefit for your participation and there is no damage because of your participation is this study. Your participation is voluntarily. You can skip any question that you don't want to answer. But in order to come up with important findings, your honest participation is important. The interview will take 20-25 minutes.

Do you agree?

Yes

No

Interviewer:

Name:

Signature:

Date:

Card No.:

**Part I: Socio-demographic information**		
**Q. No.**	**Question**	**Response**
**Q1**	Where do you live in?	1. At the town.<br>2. Out of the town
**Q2**	How long is your house from this facility?	Distance in km:<br>Distance in travelling hours:
**Q3**	How old are you?	(years)
**Q4**	What is your Religion?	1. Orthodox<br>2. Protestant<br>3. Catholic<br>4. Muslim<br>5. Others
**Q5**	What is your current educational level?	1. Illiterate<br>2. Read and write only<br>3. Primary school<br>4. Secondary school<br>5. College<br>6. University
**Q6**	Language	1. Amharic<br>2. Oromiffa<br>3. Tigregna<br>4. Guragegna<br>5. Others (please specify)
**Q7**	What is your current maritalstatus?	1. Single<br>2. Married<br>3. Divorced<br>4. Widowed<br>5. Separated
**Q8**	What is your current occupational status?	1. Student<br>2. Merchant<br>3. Government employed<br>4. Private job<br>5. Self employed<br>6. Housewife<br>7. Day labourer<br>8. Others (please specify)
**Q9**	What is your husband's occupation currently?	1. Merchant<br>2. Farmer<br>3. Government employed<br>4. Self-employed<br>5. Day labourer<br>6. Others (please specify)

**Q10**	What is your monthly income?	(in birr)
**Q11**	What is your household average expenditure in a month or annually?	Monthly (in birr):<br>Annually (in birr):

**Part II: Clinical profile**		
**Q.No.**	**Description**	**Response**
**Q12**	Date of pathological diagnosis	(dd/mm/yyyy)
**Q13**	Date of advice for breast biopsy	(dd/mm/yyyy)
**Q14**	Date of first visit at cancer hospital	(dd/mm/yyyy)
**Q15**	Total no. of visits (consultations) in TASH until diagnosis.	
**Q16**	Was patient advised to go any other place for any test?	1. Yes<br>2. No
**Q17**	Stage of breast cancer at diagnosis?	1. Stage I<br>2. Stage II<br>3. Stage III<br>4. Stage IV
**Q18**	Was FNA (fine needle aspiration cytology) peformed?<br>If yes, please specify on the findings.	1. Yes<br>2. No<br>Finding:
**Q19**	Was surgery performed?<br>If yes, when was the initial surgical treatment done?	1. Yes<br>2. No<br>Date of surgery: (dd/mm/yyyy)
**Q20**	What treatment was/ is taken?<br>If several, please indicate first treatment initiated.	1. Chemo<br>2. Hormonal
**Q21**	When was the treatment indicated in Q20 started?	(dd/mm/yyyy)
**Q22**	What was the tumor size?	1. T1m-T1b<br>2. T1c<br>3. T2<br>4. T3 and T4<br>5. Unknown
**Q23**	What was the nodal status?	1. N0<br>2. N1<br>3. N2<br>4. N3<br>5. Unknown
**Q24**	What co-morbidities illness encountered?	1. Diabetes mellitus<br>2. Stroke<br>3. Hypertension<br>4. Heart disease<br>5. No known systemic disease

**Part III: Use of alternative treatment**

**Q25**	Did you receive enough information about the importance, side effect and procedure of treatment?	1. Yes<br>2. No
**Q26**	Did you try any form of alternative treatment?	1. Yes<br>2. No
**Q27**	If yes, which one have you tried?	1. Traditional medicine<br>2. Holy water<br>3. Other (please specify):

**Part IV: Patients´perceived barriers to timely treatment initiation.**					
**Q. No.**	**Reasons for not initiate the systemic treatment timely.**	**No, not at all**	**Somewhat important**	**Important**	**Very important**
**Q28**	I thought I could handle it on my own.	0	1	2	3
**Q29**	I was concerned about what other people would think of me if I went for help.	0	1	2	3
**Q30**	I was too embarrassed or ashamed.	0	1	2	3
**Q31**	Couldn’t afford to pay for help.	0	1	2	3
**Q32**	I had no transportation, no way to get there.	0	1	2	3
**Q33**	I thought my troubles would just go away without any help.	0	1	2	3
**Q34**	I've had a bad experience with treatment before.	0	1	2	3
**Q35**	I didn't have the time.	0	1	2	3
**Q36**	I needed someone to take care of my children while I was getting help.	0	1	2	3
**Q37**	I was appointed for a long time.	0	1	2	3
**Q38**	I was scared of the side effects of the treatment.	0	1	2	3

**Part V: Multi-dimensional scale of perceived social support items questions**								
**Q.No.**	**MSPSS Items**	**Very strongly disagree**	**Strongly disagree**	**Mildly disagree**	**Neutral**	**Mildly agree**	**Strongly agree**	**Very strongly agree**
**Q39**	There is a special person who is around when I am in need.							
**Q40**	There is special person with whom I can share my joys and sorrow.							
**Q41**	My family really tries to help me.							
**Q42**	I get the emotional help and support I need from my family.							
**Q43**	I have a special person who is a real source of comfort to me.							
**Q44**	My friends really try to help me.							
**Q45**	I can count on my friends when things go wrong.							
**Q46**	I can talk about my problem with my family.							
**Q47**	I have friends with whom I can share my joys and sorrow.							
**Q48**	There is a special person in my life who cares about my feelings.							
**Q49**	My family is willing to help me make decisions.							
**Q50**	I can talk about my problems with my friends.							

Amharic Version

የውሌ ስምምነት

ውዴ ተሣታፉ

ስሜ (----) ይባሇሌ አዱስ አበባ ዩኒቨርስቲ የጤና ሣይንስ ኮላጅ ውስጥ ተማሪ የሆነችው ቤቴሌ ተሾመ በምታካሂዲው ጥናት መረጃ ሠብሣቢ ነኝ፡፡ የዚህ ቀሇ መጠይቅ ዓሊማ በጥቁር አንበሣ ካንሠር ሕክምና መስጫ ውስጥ የሚገኙ የጡት ካንሠር ታካሚዎች ሕክምናውን ሇመጀመር መሠናክሌ የሚሆኑባቸውን ነገሮች መዲሠስ ነው፡፡ በተመራማሪው የተዘጋጀውን ጥያቄ እንዱመሌሱ እርስዎ እንዯ አንደ ናሙና ሆነው ተመርጠዋሌ፡፡ ከዚህ ጥናት ሊይ የሚገኘው ውጤት ሇምርምር ዓሊማ ብቻ ይውሊሌ፡፡ የእርስዎ ስም በጥያቄ ወረቀቱ ሊይ አይሞሊም እንዱሁም እርስም የሚሠጡት መረጃ በጥንቃቄ በሚስጢር ይያዛሌ፡፡ በዚህ ጥናት ሊይ በመሣተፌ የሚዯርስና ምንም አይነት ጉዲት አይኖርም፡፡ እንዱሁም የሚያገኙት ሊሊ የተሇያ የገንዘብም ሆነ ሊሊ ጥቅማ ጥቅም አይኖርም እርስም የሚያዯርጉት ተሣትፍ በፇቃዯኝነት ሊይ የተመሠረተ ነው፡፡ መመሇስ የማይፇሌጉት ማንኛውም ጥያቄ ማሇፌ ይችሊለ፡፡ ሆኖም ትክክሇኛ ውጤት ሇማግኘት የእርስዎ እውነተኛ መረጃ መስጠትዎ አስፇሊጊ ነው፡፡ ቃሇ መጠይቁ ከ20-25 ዯቂቃ ይወስዲሌ፡፡

ይስማማለ?

አዎ

አይ

Interviewer:

Name:

Signature:

Date:

Card No.:

ክፌሌ 1: በጥናቱ ውስጥ የማሣተፈ ተካሚዎች ማኅበራዊና ስነ ሕዝብ ሁኔታ		
**ተ.ቁ**	**ጥያቄ**	**መሌስ**
Q1	የሚኖሩት የት ነው	1. ከተማ<br>2. ክ/ከተማ ውጪ
Q2	የመኖሪያ ቤትዎ ከሕክምና መስጫ ተቋሙ ምን ያህሌ ይርቃሌ?	1. በሜትር /ኪ.ሜ<br>2. በሠዓት
Q3	ዕዴሜዎ ስንት ነው?	
Q4	ሀይማኖትዎ ምንዴ ነው?	1. ኦርቶድክስ<br>2. ፕሮቴስታንት<br>3. ካቶሉክ<br>4. ሙስሉም<br>5. ላሊ /ግሇጽ
Q5	አሁን ያለበት የትምህርት ዯረጃ ምንዴ ነው?	1. ያሌተማረ<br>2. ማንበብና መፃፌ የሚችለ<br>3. የመጀመሪያ ዯረጃ ት/ርት<br>4. ሁሇተኛ ዯረጃ ትምህርት ያጠናቀቀ<br>5. ኮላጅ<br>6. ዩኒቨርሲቲ
Q6	መናገር የሚችለት ቋንቋ ምንዴ ነው?	1. አማርኛ<br>2. ኦሮምኛ<br>3. ትግርኛ<br>4. ጉራጊኛ<br>5. ላሊ /ግሇጽ/
Q7	አሁን ያለበት የጋብቻ ሁኔታ ምንዴ ነው?	1. ያሊገባ<br>2. ያገባ<br>3. የተፊታ<br>4. የሞተበት<br>5. የተሇያየ
Q8	አሁን ያለበት የሥራ ሁኔታ ምንዴ ነው?	1. ተማሪ<br>2. ነጋዳ<br>3. የመንግስት ሠራተኛ<br>4. የግሌ ሥራ<br>5. የግሌ ሥራ ተቀጣሪ<br>6. የቤት እመቤት<br>7. የቀን ሠራተኛ<br>8. ገበሬ<br>9. ላሊ /ግሇጽ
Q9	የባሇቤትዎ የሥራ ሁኔታ ምንዴ ነው?	1. ተማሪ<br>2. ነጋዳ<br>3. የመንግስት ሠራተኛ<br>4. የግሌ ሥራ<br>5. የግሌ ሥራ ተቀጣሪ

		6. የቀን ሠራተኛ<br>7. ገበሬ<br>8. ላሊ /ግሆጽ
Q10	ወርሃዊ ገቢም ምን ያህሌ ነው?	
Q11	ዓመታዊ ወይም ወርሃዊ የቤት ውጪዎ በአማካይ ምን ያህሌ ይሆናሌ	ዓመታዊ<br>ወርሃዊ

**ክፌሌ 2: የታካሚዎች የክሉኒክ መገሇጫዎች የሚገመግም ጥያቄዎች**		
**ተ.ቁ**	**መግሇጫ**	**ምሊሽ**
Q12	የበሽታው መኖር በሕክምና የተረጋገጠበት ቀን	ቀን /ወር/ዓ.ም
Q13	ሇጡት ባዮፕሲ ናሙና እንዱሠጥ የታዘዘበት ቀን	ቀን /ወር/ዓ.ም
Q14	የካንሠር ሕክምናን መጀመሪያ የሄደበት /የታዩበት ቀን	ቀን /ወር/ዓ.ም
Q15	በሽታው በሕክምና እስኪረጋገጥ ዴረስ አጠቃሊይ ወዯሕክምና በታምን ያህሌ ጊዜ ጎብኝተዋሌ፡	
Q16	ታካሚው ሇምርመራ ላሊ ቦታ እንዱሄዴ ተዯርጎ ነበር?	1. አይ<br>2. አዎ
Q17	ሇመጀመሪያ ጊዜ ሇሕክምና በቀረቡበት ጊዜ የነበረው የጡትካንሠር ዯረጃ	1. ዯረጃ I<br>2. ዯረጃ II<br>3. ዯረጃ III<br>4. ዯረጃ IV
Q18	የ FNA ምርመራ ተዯርጎ ነበር?<br>ከሆነ ውጤቱ ምን ነበር?	1. አይ<br>2. አዎ ውጤቱን መዝግብ
Q19	ቀድ ጥገና ተዯርጎ ነበር?<br>ተዯርጎ ከሆነ ቀድ ጥገናው የተካሄዯውመቼ ነበር?	1. አይ<br>2. አዎ<br>ቀን /ወር /ዓ.ም
Q20	ምን አይነት ሕክምና እየወሠደ ነው? ከአንዴ በሊይ መሌስ ሉኖርይችሊሌ	1. ኬምቴራፒ<br>2. ሆርሞናሌ ቴራፒ
Q21	ሕክምናው የተጀመረው መቼ ነበር?	ቀን /ወር /ዓ.ም
Q22	የእህጢው መጠን ምን ነበር?	1. Tim-T,b<br>2. T,C<br>3. T2<br>4. T3 T4<br>5. አይታወቅም
Q23	የእጢው ሁኔታ ምን ነበር?	1. N0<br>2. N1<br>3. N2<br>4. N3<br>5. አይታወቅም
Q24	ታካሚው ያለበት ተጨማሪ በሽታዎች	1. የስኳር በሽታ<br>2. ስትሮክ<br>3. የዯም ግፉት<br>4. የሌብ በሽታ

		5. የታወቀ ሕመም የሆም

ክፌሌ 3: የጡት ካንሠር ሕከምናን በጊዜ ከመጀመር ጋር ተያያዥነት ያሊቸው ሁኔታዎች		
Q25	ከሐኪምዎ ስሇ ሕክምናው አስፇሊጊነት፣ የጎንዮሽ ጉዲትና የአወሣሠዴ ሁኔታውን በተመሇከተ መረጃ አግኝተዋሌ?	1. አይ<br>2. አዎ
Q26	ሕክምናውን ከመጀመርዎ በፉት ሕክምና ተቋሙ ውጪ ላሊ የሕክምና አማራጮችንተጠቅመዋሌ?	1. አይ<br>2. አዎ
Q27	ከሆነ የትኛውን ሞክረዋሌ?	1. የባህሌ ሕክምና<br>2. ፀበሌ<br>3. ላሊ /ግሇጽ/

ክፌሌ 4፡ ታካሚዎች ሕክምናውን ሇመጀመር መሠናክር የሚሆኑባቸው ነገሮች					
**ተ.ቁ**	**ሕክምናውን በሰዓቱ እንዲይወስደ ያዯረቀቸው ምክንያቶች /ኬምቴሪፒ፣ ራዱዮቴራፒ፣ሆርሞናሌ ቴራፒ**	**አይ በጭራሽ**	**በተወሰነ ዯረጃ**	**አስፇሊጊ ምክንያት**	**በጣም አስፇሊጊ**
Q28	በራሴ መቆጣጠር እንዲምችሌ አስቤ ስሇነበር	0	1	2	3
Q29	የሕክምና እርዲታ በማግኘት ከሄዴኩ ሰዎች ስሇእኔ የሚኖራቸው አስተያየት ያሳስበኝ ስሇነበር	0	1	2	3
Q30	በጣም የኃፌረት ስሜት ይሠማኝ ስሇነበር	0	1	2	3
Q31	የሕክምና እርዲታውን ሇማግኘት የሚያስችሌ የገንዘብ አቅምአሌነበረኝም	0	1	2	3
Q32	ወዯ ሕክምና ቦታ ሇመሄዴ የመጓጓዣ ችግር ነበረብኝ	0	1	2	3
Q33	ያሇ ሕክምና እርዲታ በሽታው በራሱ ጊዜ የሚታወኝ መስልኝነበር	0	1	2	3
Q34	ከዚህ በፉት ከሕክምና ጋር በተያየዘ መጥፍ ሁኔታ አጋጥሞነበር	0	1	2	3
Q35	ምንም ጊዜ አሌነበረኝም	0	1	2	3
Q36	ሌጆቼን የሚንከበከብሌኝ ሰው አሌነበረም	0	1	2	3
Q37	ቀጠሮ የተሠጠኝ በጣም ሇረዠም ጊዜ ነበር	0	1	2	3
Q38	የሕክምናው የጎንዮሽ ጉዳቶችን ፈራሁ	0	1	2	3

ክፌሌ 5: የማህበራዊ ዴጋፌ ሊይ ያሇ ግንዛቤ የዘርፇ-ብዙ ዕይታ መሇኪያ								
**ተ.ቁ**	**MSPSS ጉዲዮች**	**እጅግበጣም አሌስማማም**	**አሌስማማም**	**እርግጠኛአይዯሇሁም**	**አይመሇከተኝም**	**አሊውቅም**	**እስማማሇሁ**	**እጅግበጣምእስማማሇሁ**
Q39	በምፇሌገው ጊዜ የሚዯርስሌኝ ሌዩ የሆነበጣም ጥሩ ሰው አሇ።							
Q40	ዯስታዬንና ሐዘኔን ሌጋራኝ የሚችሌ ሌዩ የሆነ በጣምጥሩ ሰው አሇ።							
Q41	ቤተሰቤ እኔን ሇመርዲት በጣም ይጥራሌ።							
Q41	የስሜት እርዲታና ዴጋፌ በምፇሌግበት ጊዜ ከቤተሰቤአገኛሇሁ።							
Q43	ጥሩ የሆነና የተዯሊዯ ሇምኞት እንዱሰማኝ የሚያዯርገኝ ሌዩ የሆነበጣም ጥሩ ሰው አሇ።							
Q44	ጓዯኞቼ እኔን ሇመርዲት በጣም ይጥራሌ።							
Q45	ችግሮቼን ከቤተሰቤ ጋር ሆኜ ሌወጣቸው እችሊሇሁ።							
Q46	ከቤተሰቤ ጋር ስሇ ችግሮቼ መነጋገር እችሊሇሁ።							
Q47	ዯስታዬንና ሐዘኔን ሊጋራቸው የምችሊቸውጓዯኞች አለኝ።							
Q48	ስሜቴን የሚረዲኝን ሌዩ የሆነ በጣም ጥሩ ሰው በሕይወቴ ውስጥ አሇ።							
Q49	ውሳኔዎችን በምወስንበት ጊዜ ቤተሰቤ ከጎኔ ሆኖ እኔን ሇመርዲት ፇቃዯኛ ነው።							
Q50	ከጓዯኞቼ ጋር ስሇ ችግሮቼ መነጋገር እችሊሇሁ።							
